# Identification of an endoplasmic reticulum stress-related signature associated with clinical prognosis and immune therapy in glioma

**DOI:** 10.1186/s12883-022-02709-y

**Published:** 2022-05-25

**Authors:** Lianxin Li, Zhihao Yang, Yinfei Zheng, Zhigang Chen, Xiaoyu Yue, Erbao Bian, Bing Zhao

**Affiliations:** 1 Department of Neurosurgery, The Second Affiliated Hospital of Anhui Medical University, Hefei, 230601 China; 2 Cerebral Vascular Disease Research Center, Anhui Medical University, 678 Fu Rong Road, Hefei, 230601 Anhui Province China

**Keywords:** Glioma, Endoplasmic reticulum stress, Gene signature, Prognosis, Function, Immune therapy

## Abstract

**Background:**

Glioma is the most common brain tumor in adults and is characterized by a short survival time and high resistance to chemotherapy. It is imperative to determine the prognosis and therapy-related targets for glioma. Endoplasmic reticulum stress (ERS), as an adaptive protective mechanism, indicates the unfolded protein response (UPR) to determine cell survival and affects chemotherapy sensitivity, which is related to the prognosis of glioma.

**Methods:**

Our research used the TCGA database as the training group and the CGGA database as the testing group. Lasso regression and Cox analysis were performed to construct an ERS signature-based risk score model in glioma. Three methods (time-dependent receiver operating characteristic analysis and multivariate and univariate Cox regression analysis) were applied to assess the independent prognostic effect of texture parameters. Consensus clustering was used to classify the two clusters. In addition, functional and immune analyses were performed to assess the malignant process and immune microenvironment. Immunotherapy and anticancer drug response prediction were adopted to evaluate immune checkpoint and chemotherapy sensitivity.

**Results:**

The results revealed that the 7-gene signature strongly predicts glioma prognosis. The two clusters have markedly distinct molecular and prognostic features. The validation group result revealed that the signature has exceptional repeatability and certainty. Functional analysis showed that the ERS-related gene signature was closely associated with the malignant process and prognosis of tumors. Immune analysis indicated that the ERS-related gene signature is strongly related to immune infiltration. Immunotherapy and anticancer drug response prediction indicated that the ERS-related gene signature is positively correlated with immune checkpoint and chemotherapy sensitivity.

**Conclusions:**

Collectively, the ERS-related risk model can provide a novel signature to predict glioma prognosis and treatment.

**Supplementary Information:**

The online version contains supplementary material available at 10.1186/s12883-022-02709-y.

## Introduction

Malignant glioma is the most common primary brain tumor in humans and is histologically classified as diffuse astrocytoma (grade II), anaplastic astrocytoma (grade III), and glioblastomas (grade IV) [1, 2]. Gliomas are classified into circumscribed gliomas (WHO grade I) and diffusely infiltrating gliomas (WHO grades II-IV) according to their pattern of growth and the presence or absence of the IDH mutation [3]. Current treatment approaches for glioma mainly include surgery, chemotherapy, and radiotherapy [4]. Immunotherapy has also shown excellent prospects in preclinical studies [5–7]. Although significant advances have been made in the surgery of malignant gliomas, complete resection is impractical [8]. With its multichemotherapy resistance and limited clinical application of immunotherapy, recurrence is almost unavoidable, and the median survival time of glioma is only 12–15 months.

Recent studies reported that endoplasmic reticulum stress (ERS) could affect the prognosis and treatment of glioma [9, 10]. As an adaptive protective mechanism, endoplasmic reticulum stress has been reported in many studies to play a critical role in the prognosis of glioma [11–14]. ER stress can trigger cells to activate a series of adaptive responses called the unfolded protein response (UPR), which involves a series of protein signaling pathways triggered by different perturbations in the general response of the endoplasmic reticulum (ER) that direct the upregulation of a series of gene functions, such as protein folding, quality control, and secretion. The UPR has evolved to establish a complex network of interconnected signaling pathways, and three distinct types of signal transducers have been defined in the UPR, including inositol-requiring protein-1 (IRE1), activating transcription factor-6 (ATF6), and protein kinase RNA (PKR)-like ER kinase (PERK) signaling pathways [15]. Generally, the UPR alleviates ER stress by arresting general translation, degrading misfolded proteins, and upregulating chaperones and folding enzymes. However, under intense or constant endoplasmic reticulum stress, the UPR not only induces cell apoptosis [16–18] and regulates chemotherapeutic sensitivity [19–22] but also modulates the immune microenvironment and elicits immunogenic cancer cell death(ICD) [23, 24]. Therefore, identifying ERS-related genes, establishing related prognostic signatures, and evaluating the immune microenvironment and checkpoints will improve the prognosis and treatments of glioma patients.

Recently, the comprehensive mutational landscape for the major glioma types was shown by genome-wide molecular profiling analyses [25–27]. Molecular characteristics can extend the prognosis of glioma patients and allow for the exploration of more precision treatments [27]. Based on this, our study used The Cancer Genome Atlas (TCGA) database as the training dataset and the Chinese Glioma Genome Atlas (CGGA) database as the testing dataset to search the clinical value of the ERS-related gene signature. First, Cox regression and Lasso regression were performed to identify whether the ERS-related gene signature was correlated with significant overall survival (OS) of glioma patients. We also applied the Kaplan–Meier (KM) estimator and the receiver operating characteristic (ROC) algorithm to validate the accuracy of the signature. Then, we used consensus clustering analysis to evaluate differences in molecular characteristics. Furthermore, Gene Set Enrichment Analysis (GSEA), Gene Ontology (GO), and Kyoto Encyclopedia of Genes and Genomes (KEGG) pathway functional annotation methods were performed to further explore the role of the ERS-related gene signature in glioma. In addition, we evaluated the immune microenvironment of patients and predicted the immune checkpoint and chemotherapy sensitivity to explore the therapeutic response. Taken together, our results indicated that the ERS-related gene signature will be better for evaluating glioma prognosis and predicting novel targets in glioma treatment.

## Materials and methods

### Source of data

**Fig. 1:**
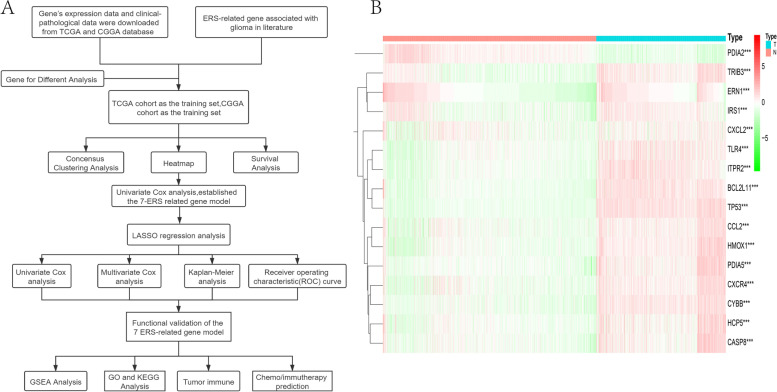
**|** The total workflow and sixteen ERS-related genes differentiated from the TCGA database. (**A**) The wrkflow of this study: data collection, analysis, and validation. (**B**) The heatmap shows 16 ERS-related genes that were obviously different between the tumor and normal groups. **P < 0.01; ***P < 0.001

Endoplasmic reticulum stress gene expression profiles and 689 glioma corresponding samples’ clinical data, such as age, sex, cancer type, and survival time, were obtained from the TCGA database (https://xena.ucsc.edu), 1136 normal samples were obtained in GTEx, and 1018 glioma clinical samples were obtained from the CGGA database (http://www.cgga.org.cn/index.jsp). All of the data with incomplete clinical information were filtered. A total of 169 endoplasmic reticulum stress-related genes were quoted from classic literature reviews. Then, the difference analysis of genes was performed by the R program (http://cran.r-project.org) to screen the intersecting genes. We identified these differentially expressed genes between glioma and normal samples, set logFC value <− 1 or > 1, *p* value< 0.001, and 16 significant ERS-related genes were ultimately selected. The study’s workflow is shown in Fig. 1.

### Bioinformatics analysis

Next, we applied univariate Cox regression in the TCGA dataset and selected *P* < 0.01 to screen 13 significant genes. Consistent univariate Cox regression in the CGGA dataset selected *P* < 0.01 to screen 10 significant genes. Then, 7 ERS-related genes were used as candidates for the prognostic signature by the Lasso regression algorithm. HR < 1 was considered a protective factor, whereas HR > 1 was considered a risk factor. To calculate the risk score of each glioma patient, multivariate regression analysis was performed to evaluate the relative contribution of candidate genes of the prognostic signature. The formula was as follows:

$$\mathrm{Risk}\ \mathrm{score}={\sum}_{\mathrm{x}=1}^{\mathrm{n}}\;\mathrm{Coef}\;\mathrm{x}\ast \mathrm{YX}$$

Coef(i) and X(i) represent ERS-related gene expression values and regression coefficients. The Lasso regression algorithm by R programming language was performed to calculate the risk score. The regression coefficient calculated by the linear combination of multiple genes divided subjects into high-risk and low-risk subtypes by median risk score. Kaplan–Meier analyses evaluated the relationship between different subtypes and survival rates. To evaluate the predictive prognosis accuracy of the 7-gene signature, ROC curves were adopted to assess the sensitivity and specificity using the “survival ROC” package.

### Consensus clustering and survival analysis

The R program package “Consensus Cluster Plus” was used for stratification. We performed a consensus matrix and cumulative distribution function (CDF) to assess the optimal cluster amounts. Then, we utilized the Kaplan–Meier curve to calculate the survival curve drawn by the R packages Survival and Survminer. This relationship was validated using a log-rank test, and the results revealed the correlation between the different genes and overall patient survival.

### Functional analysis

We performed the correlation algorithm from the function “cor. Test” with R to identify the genes with a Spear correlation value < 0.4 or > 0.4, *p* < 0.001. The hallmark datasets were obtained from the Molecular Model Database (MMDB). We adopted gene set enrichment analysis (GSEA) to determine the signaling pathways, and the seven-ERS-related gene signature calculated the results by running GSEA software (4.1.1). In addition, we adopted the Kyoto Encyclopedia of Genes and Genomes (KEGG) algorithm [28] and Gene Ontology (GO) algorithm by the R package “clusterprofiler” [29] with the R language program to assess the cell functions related to risk factors for signature-based on TCGA database. The packages “ggplot2” and “pheatmap” were used for visualization.

### Immune analysis

We adopted the R language “ESTIMATE” package to assess the immune-stromal component of each sample in the TME, and it is presented as three scores: immune, stromal, and estimate scores. According to these results, tumor purity was calculated, and the interaction between them and the expression of ERS-related genes was calculated. The RNA sequencing data and published enrichment of immune cell metagenes were used as input and then scored by single-sample gene set enrichment analysis (ssGSEA) [30] to calculate the infiltration degree of 24 immune cells in all samples [31]. The “GSVA” R package was implemented to estimate the z scores of a gene set over the samples [32]. After deconvolution, we mapped the estimated proportions to actual cell types in the mixtures. Then, we adopted Pearson’s test to evaluate the correlations between immune cell abundance and ERS-related gene expression, and “heatmap” and “vioplot” were used to visualize the results by R language.

### Prediction of chemotherapeutic and immunotherapeutic responses

We performed subclass mapping [33] and tumor immune dysfunction and exclusion (TIDE) algorithms in the TCGA dataset to assess the clinical immune checkpoint response between the high-risk subtype and the low-risk subtype. We used the Pharmaceutical Sensitivity Genomics in Cancer (GDSC) dataset (https://www.cancerrxgene.org/) to assess each sample’s chemotherapeutic response. Based on the GDSC data, we adopted the half-maximal inhibitory concentration (IC50) to estimate the drug response. The forecasting program was assumed by the package “pRRophetic” in R, which was applied for 10-fold cross-validation and other parameters by default [34].

### Analysis of data

We used the GraphPad Prism 8 program for statistical analysis and graphing. The RStutio program performed multivariate and univariate Cox regression analyses. ROC and Kaplan–Meier curves were generated to verify the reliability of the risk model. The chi-square test was used to determine clinical feature differences among samples classified by median risk score. We also used Student’s t-test, and we assessed correlations by using Pearson’s correlation analysis. A 2-tailed *p* value< 0.05 was defined as statistically significant.

## Results

### The total workflow and identification of ERS-related genes

We downloaded all gene expression, clinical and pathological data of glioma patients from the TCGA dataset. All of the clinical data and acquired expression data were examined to determine acceptability, and 670 glioma patients reported relevant prognostic information. A set of pretreatments was applied to the acquired expression data of relative genes from all the glioma samples. These methods include searching for various gene expressions associated with ERS, supplementing missing information, and deleting cases with incomplete data. The genes associated with endoplasmic reticulum stress (ERS) pathways were obtained from the classic literature. Then, we established the ERS-related gene signature, evaluated the survival and prognosis of patients, performed multivariate analysis, and verified functional analysis and prediction for chemo/immunotherapy based on it (Fig. 1A).

Next, differential analysis was performed to analyze differentially expressed genes between the tumor and normal sets. With the filter conditions of *p* value< 0.001 and correlation coefficients> 1 or < − 1, Pearson’s correlation analysis was performed between the 169 gene expression levels in samples to acquire ERS-related genes. Then, we carried out a differential analysis between normal and tumor samples by the R program (http://cran.r-project.org). Sixteen ERS-related genes, including CXCL2, ERN1, IRS1, HCP5, CASP8, CCL2, TLR4, TRIB3, PDIA5, CXCR4, HMOX1, ITPR2, CYBB, PDIA2, BCL2L11, and TP53, showed significant differences between the two clusters. Among them, fifteen were highly expressed in tumor tissues (TRIB3, ERN1, IRS1, CXCL2, TLR4, ITPR2, BCL2L11, TP53, CCL2, HMOX1, PDIA5, CXCR4, CYBB, HCP5, CASP8), and one was highly expressed in normal tissues (PDIA2) (Fig. 1B and Table 1).

**Table 1:** Full names and *p*-value of the 16 genes associated with ERS

Gene	Full Name	*p*-value
CXCL2	C-X-C motif chemokine ligand 2	6.81E-25
ERN1	endoplasmic reticulum to nucleus signaling 1	1.47E-56
IRS1	insulin receptor substrate 1	8.32E-57
HCP5	HLA complex P5	1.54E-74
CASP8	caspase 8	1.22E-78
CCL2	C-C motif chemokine ligand 2	5.73E-119
TLR4	toll like receptor 4	3.54E-120
TRIB3	tribbles pseudokinase 3	4.32E-129
PDIA5	protein disulfide isomerase family A member 5	9.68E-131
CXCR4	C-X-C motif chemokine receptor 4	1.15E-141
HMOX1	heme oxygenase 1	1.80E-184
ITPR2	inositol 1,4,5-trisphosphate receptor type 2	4.68E-189
CYBB	cytochrome b-245 beta chain	6.05E-198
PDIA2	protein disulfide isomerase family A member 2	6.05E-228
BCL2L11	BCL2 like 11	2.37E-243
TP53	tumor protein p53	2.33E-267

### Establishment of the ERS-related gene signature

**Fig. 2:**
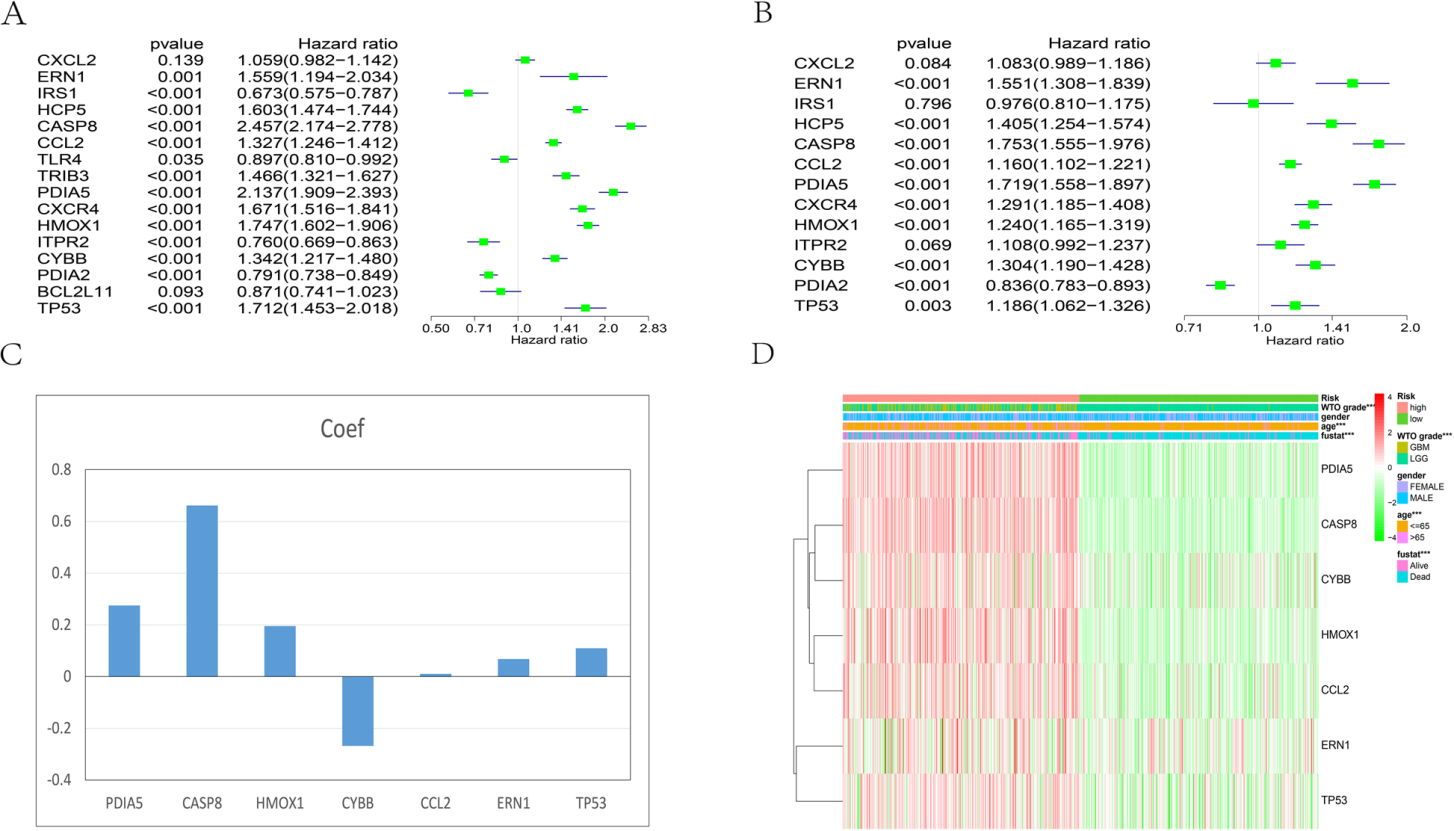
Construction of the 7-gene risk model by the Lasso regression algorithm. (**A**) The *p* values and hazard ratios of 16 ERS-related genes in the TCGA set. (**B**) *p* values and hazard ratios of 13 ERS-related genes in the CGGA set. (**C**) Coefficient values of 7 screened genes. (**D**) Heatmap showing the correlation of clinicopathological features and risk scores. ***P < 0.001

We utilized the TCGA dataset as the training group to calculate the prediction value of the risk score model. To construct the ERS-related gene signature, we first adopted univariate Cox regression analysis to screen genes in the training cohorts and testing cohorts, and 10 genes were chosen as risk coefficients because these 10 genes had a significant *p* value in the TCGA set and CGGA set (Fig. 2A-B). Then, we identified seven genes as meaningful covariates to gauge the prognosis rate (Fig. 2C). Next, we utilized the gene expression and correlation coefficients to calculate the patients’ risk scores. According to the median risk score, the training dataset was segmented into a high-risk subtype and a low-risk subtype to assess the accuracy of the risk score, which served as a factor to evaluate characteristic genes. There were significant differences in clinical and molecular features between the high- and low-risk sets (Table 2). The high-risk set was related to age, survival state, and glioma grade (Fig. 2D).

**Table 2:** Glioma samples’ relevance among 7-gene-based risk-score and clinicopathological elements in two databases. The training and testing RNA-seq set come from the TCGA and CGGA database. Ns: no significance; Bold type statistics show a significant difference (*P* < 0.05)

Training set RNA-seq cohort (*n* = 661)			
	Low-risk score	High- risk score	
Features	*n* = 333	*n* = 332	*P*- value
Age			
≤ 65	316	259	< 0.0001
>65	16	70	
Gender			
Male	180	202	ns
Female	152	127	
Grader			
LGG	328	175	< 0.0001
GBM	4	154	
Vital status			
Alive	48	171	< 0.0001
Dead	284	158	
Testing set RNA-seq cohort(*n* = 465)			
	Low-risk score	High- risk score	
Features	*n* = 165	*n* = 115	*P*- value
Age			
≤ 65	189	255	< 0.05
>65	3	18	
Gender			
Male	102	164	ns
Female	90	109	
Grader			
LGG	164	135	< 0.0001
GBM	28	138	
Vital status			
Alive	125	76	< 0.0001
Dead	67	197	

In the CGGA dataset, we calculated the risk score for each sample to determine whether the characteristics of the ERS-related gene risk model had the same applicability. Similarly, the high-risk group had more malignant clinical characteristics, including IDH mutation, WHO grade, age, fustat, 1p19q noncodel, and recurrence, than the low-risk group (Fig. S1A-G).

### The 7-gene signature has exceptional predictive power

**Fig. 3:**
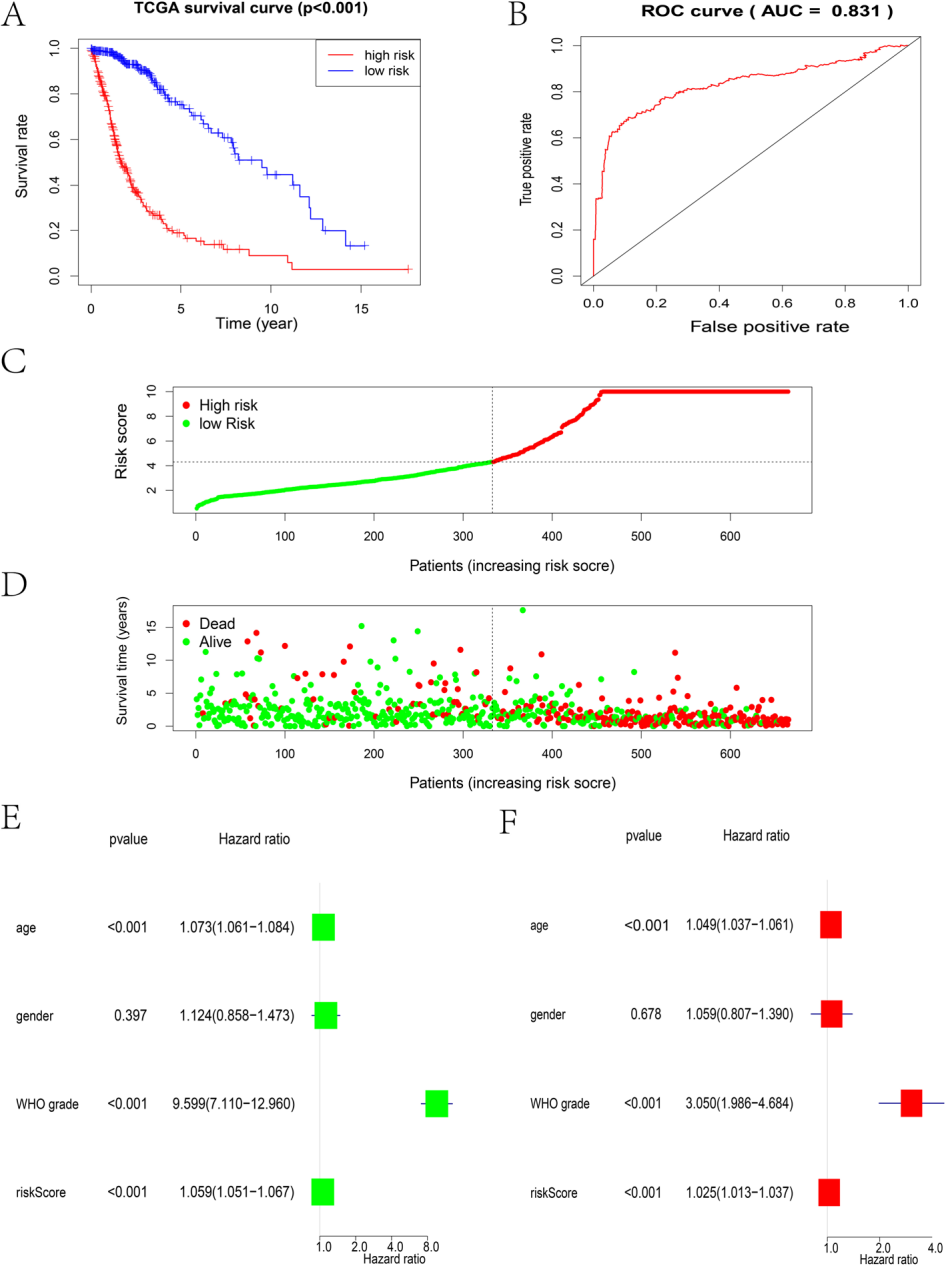
Prediction of the 7-gene signature of glioma patients in the TCGA and CGGA datasets. (**A**) Kaplan–Meier algorithm among the high-risk group and low-risk group from the TCGA dataset. (**B**) The ROC algorithm indicates the specificity and sensitivity to predict survival based on the ERS-related signature from the TCGA database. (**C**) The risk curve represents the risk score and distribution of 665 cases from the TCGA database. (**D**) The survival status graph shows the difference in survival time of 665 cases from the TCGA database (each point represents a sample, C-D). (**E**) Univariate Cox regression algorithm of clinical and pathological features for survival rate from the TCGA dataset. (**F**) Multivariate Cox regression algorithm of clinical and pathological features for survival rate from the TCGA dataset. **P < 0.01; ***P < 0.001

The Kaplan–Meier curve shows that low-risk subtype patients have a remarkably longer overall survival rate (Fig. 3A). Additionally, we used the ROC curve to evaluate the sensitivity and precision of the risk score by analyzing areas under the curve of the AUCs. The AUC value was 0.831, which indicated its strong predictive power (Fig. 3B). Next, the 7-gene risk model was applied to assess glioma sample survival status and survival time. The results showed the distribution of risk scores and survival states of the 7-gene risk model (Fig. 3C-D). We performed multivariate Cox regression and univariate Cox regression methods to determine whether the risk coefficient for glioma prognosis can be a significant prognostic element. The risk score was not associated with sex but was strongly associated with the patient’s age, grade, and risk score, which could be a significant prognostic element (Fig. 3E-F). In univariate Cox regression, the hazard ratio (HR) along with the 95% CI was 1.059 (1.051–1.067) (*P* < 0.001) (Fig. 3E) and 1.025 (1.013–1.037) (*P* < 0.05) in multivariate Cox regression analyses (Fig. 3F).

Consistent results were shown in the validation set. The overall survival (OS) results revealed that low-risk subtype patients also had a higher overall survival rate (Fig. S1H). The ROC curve exhibited great sensitivity and precision for survival predictions, and the AUC value was 0.74 (Fig. S1I). Multivariate and univariate Cox regression analyses were adopted to prove that the risk score can be a significant prognostic element (Fig. S1 J-K). The hazard ratio (HR) of the risk score and 95% CI were 1.865 (1.663–2.093) (*P* < 0.001) in univariate Cox regression analyses (Fig. S1J) and 1.437 (1.262–1.636) (*P* < 0.05) in multivariate Cox regression analyses (Fig. S1K).

### Consensus clustering of the ERS-related signature

**Fig. 4:**
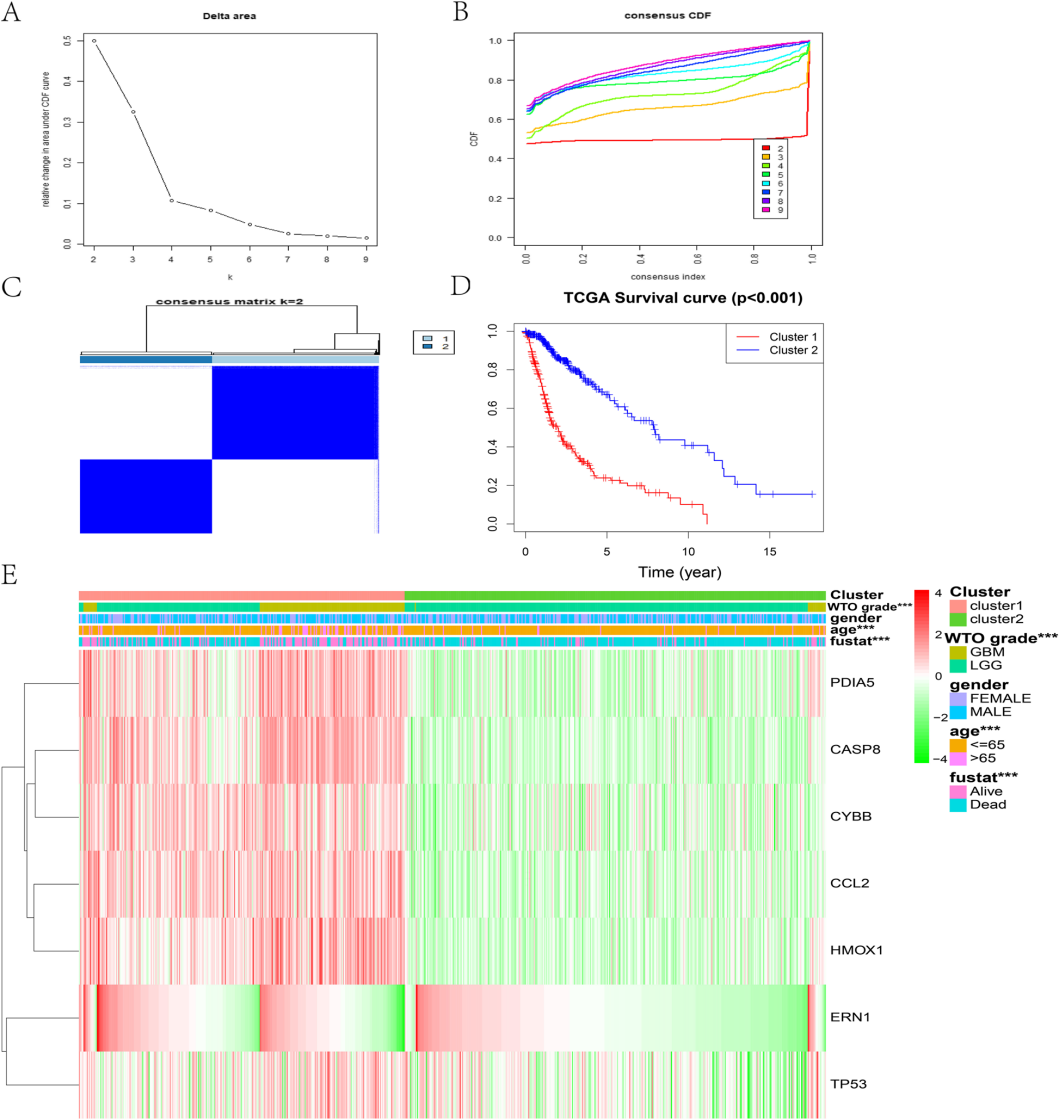
The ERS-related gene signature could identify the molecular and clinical features based on glioma patients. (**A**) The relative changes in the area under the CDF curve (k = 2 to 9). (**B**) Consensus clustering matrix for k = 2. (**C**) Consensus clustering matrix for k = 2 of 689 glioma samples from the TCGA database. (**D**) The cluster 1 and cluster 2 patients for survival curve based on TCGA clinical data. (**E**) Heatmap of ERS-related genes among the two clusters based on the TCGA dataset. CDF, cumulative distribution function; ***P < 0.001

According to the 7-gene signature, a consensus cluster of 689 samples classified two clusters based on the TCGA cohort. The stability of the cluster increased from k = 2 to 9 (Fig. 4A-C). Cluster 1 was significantly related to longer overall survival (OS) (Fig. 4D). In addition, the results indicated that the two clusters had significantly different molecular and clinical features. For the training set, cluster 1 was correlated with age, grade, and survival state (*p* < 0.001) (Fig. 4E). Consistently, the result was validated in the testing cohort (Fig. S2A-G). The CGGA testing set demonstrated a similar distinction between the two subtypes (Fig. S2F). The above results revealed that the ERS-related gene signature is related to the survival rate of patients with glioma.

### Functional analysis of the 7-gene signature

**Fig. 5:**
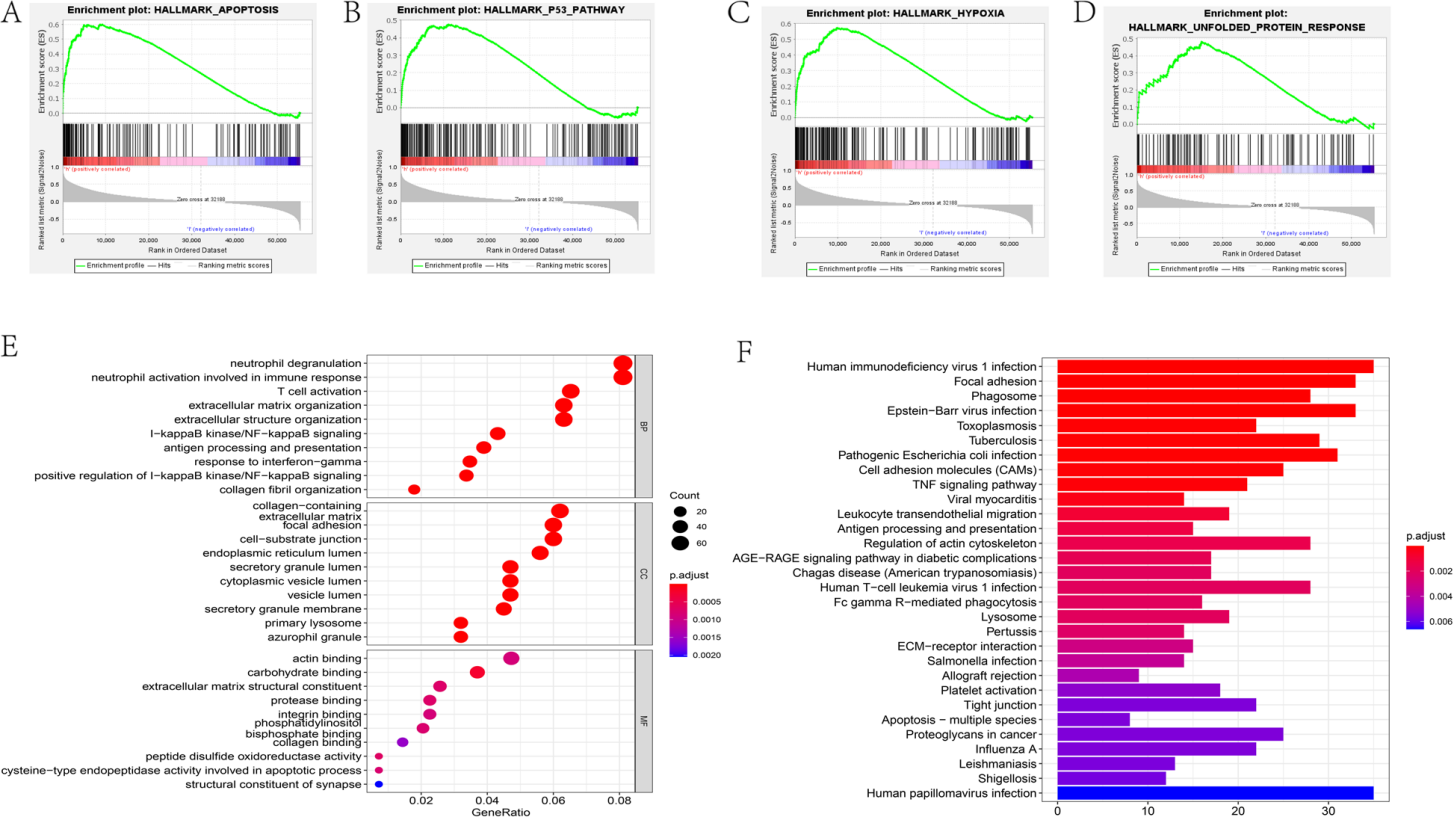
Functional analysis based on the 7-gene signature. (**A-D**) GSEA showed that the signature was enriched in 4 pathways in hallmarks. (**E**) GO analysis based on 4400 genes was strongly related to the 7-gene signature. (**F**) KEGG analysis was related to the risk score

To assess 7-gene signature characteristics of functional changes, we adopted gene set enrichment analysis (GSEA) between high-risk and low-risk sets. We found that the high-risk set had a strong relation to apoptosis, the p53 pathway, the unfolded protein response, and hypoxia compared with the low-risk set (Fig. 5A-D). In addition, we used the GO algorithm to assess the relevance among different functional pathways in the high-risk and low-risk sets. Consistently, it was found that the signature was mainly enhanced in NF-kappaB signaling, endoplasmic reticulum lumen, and integrin binding (Fig. 5E). Furthermore, the KEGG algorithm revealed that the high-risk set was correlated with cell adhesion molecules (CAMs), the TNF signaling pathway, and apoptosis-related processes (Fig. 5F), all of which are closely associated with tumor angiogenesis, malignant processes and the development of the immune microenvironment. The gene signature was correlated with the above processes, resulting in a worse prognosis for glioma patients.

### ERS-related gene signature correlates with tumor immunity in glioma

**Fig. 6:**
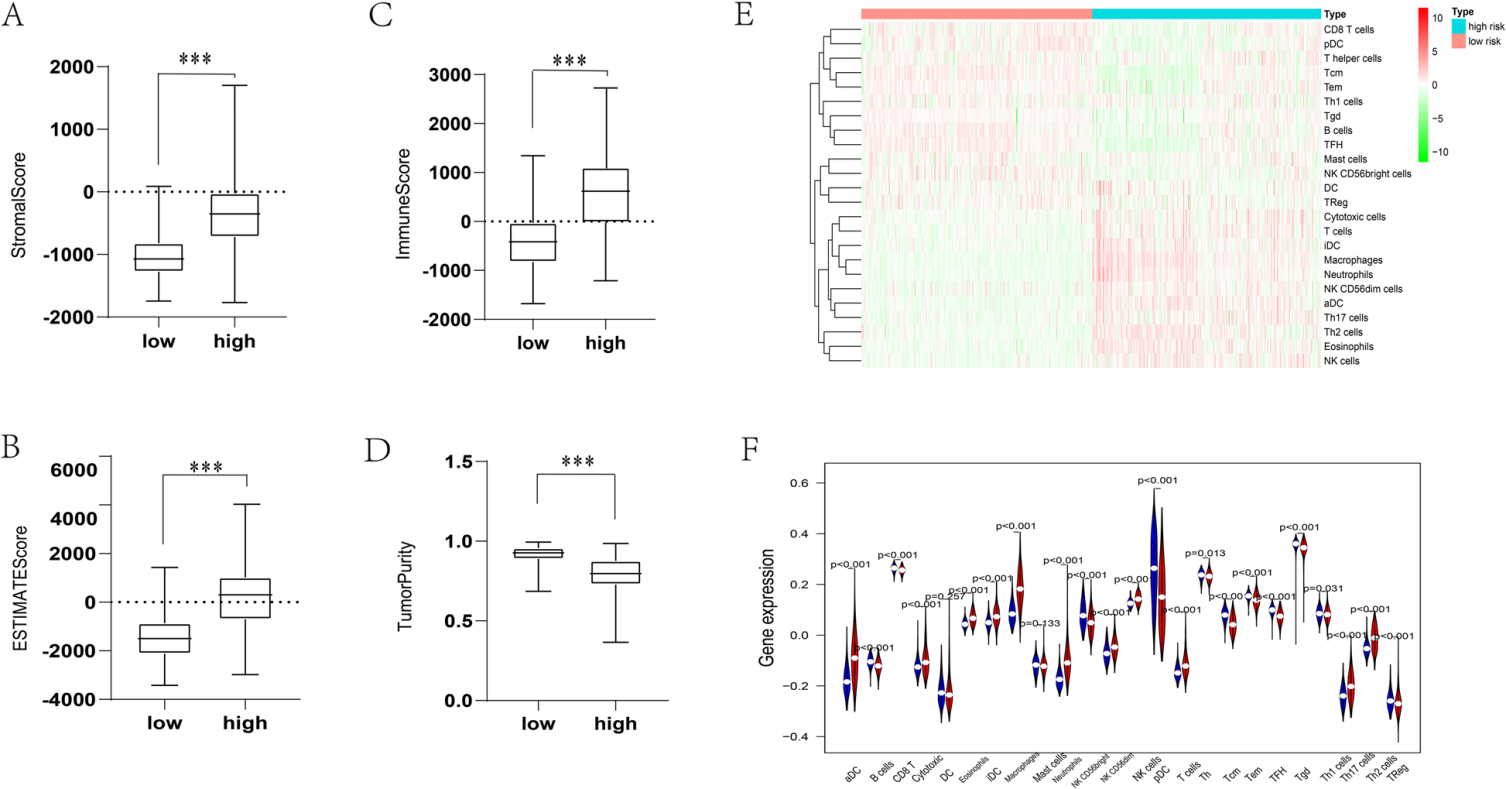
Correlation between the risk of glioma and the immune infiltration of glioma patients. (**A-D**) Immune score, ESTIMATE score, stromal score, and tumor purity were compared between the high-risk group and the low-risk group. The p value was calculated with the two-sided Wilcoxon rank-sum test. (**E**) Heatmap of the tumor infiltration proportions in the glioma immune microenvironment cells quantified by ImmuCellAI. (**F**) The relative percentage of TIICs for the high-risk subtype and low-risk subtype in glioma. ***P < 0.001

Previous studies have revealed that tumor-infiltrating immune cells (TIICs) are broadly dispersed in the tumor microenvironment (TME) and affect the disparate phase of tumor evolution. The stromal score and immune score in the TME represent the different proportions of stromal and immune cells [35]. High-risk and low-risk groups were divided based on the median value of the risk score in the TCGA profiles. According to the ssGSEA algorithm, we established 24 immune cell subtypes to assess the glioma samples’ immune and stromal microenvironment and evaluated the interaction among them and the ERS-related gene signature. The analysis revealed that the ERS-related gene signature was significantly associated with the immune score, stromal score, and estimate score but was negatively related to tumor purity (Fig. 6A-D) because immune cells comprised the bulk of nontumor ingredients in the microenvironment. Generally, the results mentioned above revealed that the ERS-related gene signature was positively related to the immune scores of glioma cells. In addition, we assumed the abundance of TIICs to evaluate the relationship of the immune microenvironment and gene signature. The proportion of the immune cell fraction is shown in Fig. 6E. We observed that the ERS-related gene signature was related to a high percentage of aDC cells, eosinophil cells, iDC macrophages cells, neutrophils cells, NK cells, Th17 cells, T cells, and Th2 cells (*P* < 0.001); among them, aDC cells showed the strongest correlation. Conversely, B cells, CD8 T cells, NK CD56 cells, pDC cells, Tcm cells, Tem cells, and TFH cells were related to lower expression of the ERS-related gene signature (*P* < 0.001); among them, pDC cells had the strongest relationship (Fig. 6E-F). Collectively, these results indicated that the ERS-related gene signature has a strong relation to immune infiltration, directs various tumor immune microenvironment types, and may play a role in the glioma malignant process.

### Prediction for immunotherapy and anticancer drug response

**Fig. 7:**
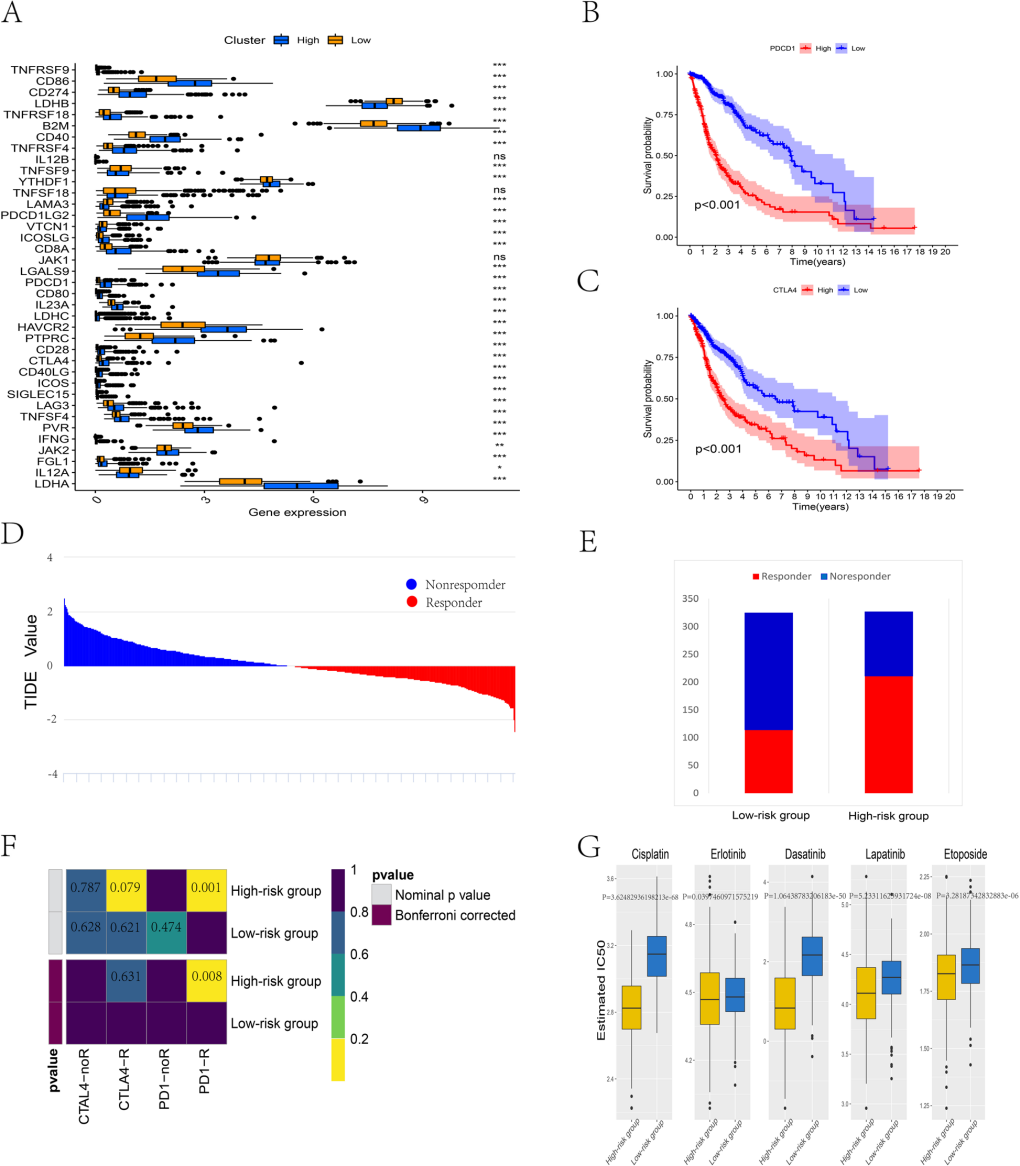
Differential putative chemotherapeutic and immunotherapeutic response. (**A**) The response of 38 immune checkpoints among the high- and low-risk sets in glioma. (**B**) Survival curve of PDCD1 (PD1) among high- and low-risk subtypes. (**C**) Survival curve of CTLA4 between high- and low-risk subtypes. (**D-E**) The TIDE value and response results to immunotherapy of patients with glioma. (**F**) The Submap algorithm showed that the high-risk set was more sensitive to CTAL-4 and anti-PD-1 therapy (Bonferroni-corrected *P* < 0.05). (**G**) Estimated IC50 shows the efficiency of chemotherapy to the high- and low-risk sets by cisplatin, erlotinib, dasatinib, lapatinib, and etoposide; ***P < 0.001

By measuring 38 immune checkpoints and calculating the survival rates, we found that the high-risk subtype showed a greater response and shorter survival time to PDCD1 (PD-1) and CTLA4 checkpoints (Fig. 7A-C). Then, we performed subclass mapping analysis to assess the immunotherapy response from the TCGA glioma dataset. Although immune checkpoint-targeted medicine did not show common benefits to glioma, the results indicated that compared to the low-risk set, the high-risk set responded better to immunotherapy (*P* = 3.606e-14) (Fig. 7D-E). We adopted subclass mapping for the TIDE algorithm to correlate the ERS-related gene signature expression files with an announced dataset with 47 melanoma patients who reacted to the immunotherapeutic response. The analysis showed that the high-risk set had the most encouraging response to PD1 treatments (Bonferroni correction *P* < 0.05) (Fig. 7F).

Chemotherapy is the most traditional remedy for glioma. We assessed the reaction of two clusters. The prediction signature of the Genomics of Drug Sensitivity in Cancer (GDSC) cell line database was trained by ridge regression. We used 10-fold cross-validation for the TCGA set to assess satisfactory prediction precision. In the TCGA dataset, each sample’s IC50 was analyzed by the predictive signature of chemo-drugs. There was a significant difference against the low-risk group of estimated IC50 for several drugs. The high-risk set that adopted chemotherapy seemed to express more sensitivity (cisplatin, erlotinib, dasatinib, lapatinib, and etoposide, *P* < 0.05) (Fig. 7G).

### Prognostic potential of seven genes in glioma patients

**Fig. 8:**
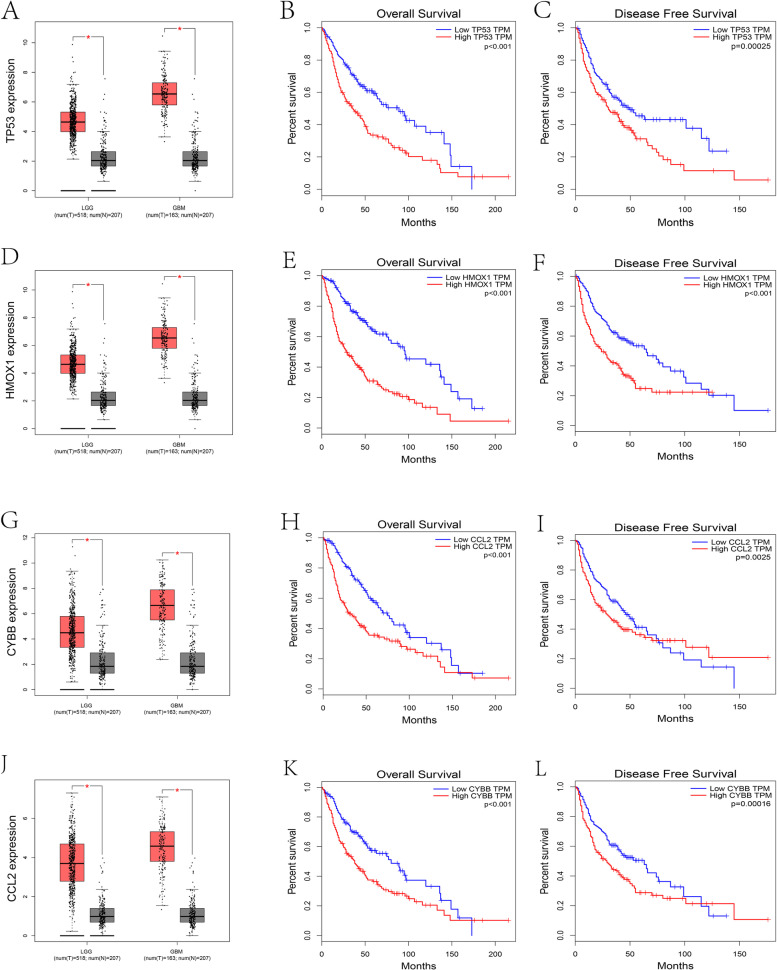
TP53, HMOX1, CCL2, and CYBB were selected from the seven-ERS-related gene signature. (**A**) Differences in TP53 expression between the tumor and normal groups from the GTEX and TCGA databases. (**B-C**) Kaplan–Meier overall survival and disease-free survival curves showed the correlation between TP53 expression and survival rate based on the TCGA dataset. (**D**) Differences in HMOX1 expression between the tumor and normal groups from the GTEX and TCGA databases. (**E-F**) Kaplan–Meier overall survival and disease-free survival curves showed the correlation between HMOX1 expression and survival rate based on the TCGA dataset. (**G**) Differences in CCL2 expression between the tumor and normal groups from the GTEX and TCGA databases. (**H-I**) Kaplan–Meier overall survival and disease-free survival curves showed the correlation between CCL2 expression and survival rate based on the TCGA dataset. (**J**) Differences in CYBB expression between the tumor and normal groups from the GTEX and TCGA databases. (**K-L**) Kaplan–Meier overall survival and disease-free survival curves showed the correlation between CYBB expression and survival rate based on the TCGA dataset. ***P < 0.001

We analyzed the gene expression data from the normal and tumor groups in different databases, and it was revealed that 7 genes were significantly different except for ERN1. Then, we analyzed the seven genes’ disease-free survival and overall survival time among the two expression sets based on the TCGA database, and high expression set patients were related to a poor prognosis (Fig. 8 and Fig. S3).

## Discussion

Although surgery and chemotherapy have exact effects, recurrence is a considerable challenge in glioma treatment. It is critical to identify the prognosis and therapeutic-related targets of glioma. Recently, the TCGA glioma database has been used beyond targets of oncogenesis and has potential prognostic value. Additionally, immune-related genes have been proven to evaluate glioma prognosis [36–38]. Because the UPR not only induces cell apoptosis [16–18] and regulates chemotherapeutic sensitivity [19–22] but also modulates the immune microenvironment and elicits immunogenic cancer cell death(ICD) [23, 24], we assumed that detecting ERS-related genes is incredibly significant to evaluate prognosis and the immune microenvironment, contributes to finding prognostic targets and develops more effective therapy sites for glioma. Therefore, we identified 7 genes associated with ERS in gliomas, each of which has independent functions associated with ERS to affect glioma prognosis [39–47]. In addition, integrating multiple gene biomarkers into a single signature was adopted because the prediction accuracy of the signature can be enhanced compared to a single biomarker [48]. Previous studies have reported that this biomarker signature of gliomas, such as the autophagy-related signature [49, 50], ferroptosis-related signature [51], and mesenchymal-related signature [52], has shown strong prognostic power. In addition, although an ERS-related signature has been reported, it is entirely different from ours, and too many genes to make up the signature results inconvenient and impractical in clinical application. We quoted 169 ERS-related genes from classic literature and screened 7 genes to construct the signature, which demonstrated excellent advantages for the accuracy and efficiency of prediction.

Therefore, we examined the gene profiles from TCGA and CGGA glioma databases [53]. Then, Lasso regression and Cox analysis [54] were performed to construct an ERS signature-based risk score model for glioma. The 7-gene signature could divide glioma patients into high- and low-risk subtypes. There were noticeable clinical differences between the high- and low-risk subtypes, such as survival, age, and WHO grade. Three methods (time-dependent receiver operating characteristic analysis and multivariate and univariate Cox regression analysis) [55] were adopted to evaluate the independent prognostic effect of texture parameters. Low-risk patients had a longer survival time than high-risk patients, regardless of the training set or the validation set, and the AUC value was 0.7 or more. The 7-gene gene signature has strong predictive power for glioma prognosis. In addition, the consensus clustering algorithm was used to classify two clusters, and the results showed that the two clusters had significant molecular and prognostic features, as reported previously [56]. Next, in the validation set, we demonstrated the prognostic value of the signature, similar to the training set, and the results showed that the signature had excellent repeatability and precision. Collectively, the ERS-related risk model can provide a novel signature to evaluate the prognosis and treatment of glioma.

Our results indicated that the signature in GSEA showed more enrichment in the biological pathways of unfolded protein response, apoptosis, P53 pathway, hypoxia, and PI3K AKT mTOR signaling. The UPR alleviates ER stress by arresting general translation, degrading misfolded proteins, and upregulating chaperones and folding enzymes. However, when there is intense or constant ER stress, the UPR induces apoptosis, resulting in cell death [16]. On the one hand, the TP53 tumor suppressor gene arrests cells in G0/1 or triggers apoptosis in response to genotoxic stress [56]; on the other hand, the P53 signaling pathway significantly upregulates the progression of glioma [57]. Through the activation of transcription factors, hypoxia induces either direct or indirect changes in the biology of glioma and its microenvironment, leading to increased aggressiveness and glioma resistance to therapy [58]. Hypoxia-induced PLOD2 promotes glioma cell migration and invasion via modulation of the PI3K/AKT signaling pathway and promotion of EMT [59]. In addition, the results revealed that the ERS-related gene signature in the GO and KEGG analyses was closely related to antigen processing and presentation, T-cell activation, integrin binding, cell adhesion molecules (CAMs), endoplasmic reticulum lumen, and apoptosis-related processes. Many strategies associated with T-cell activation are in clinical development: anticancer vaccines aiming at using glioma antigens to induce tumor-specific effector T cells and memory T cells are able to reduce the ability to control tumor mass [60]. Experimental research shows interesting adoptive antigen-specific T-cell therapy results for glioma [61]. ERS can trigger apoptosis when high levels of unfolded proteins are persistent [62–64], including three apoptotic pathways: (1) activation of CHOP, (2) c-Jun N-terminal kinase (JNK)-mediated apoptosis activation and (3) activation of ER-associated caspases. Integrins are a family of cell surface molecules of the cell–cell and cell-extracellular matrix, which are involved in essential cellular processes such as adhesion, migration, invasion, and angiogenesis [65]. The above processes are strongly associated with malignant cancer processes, including tumor angiogenesis and progression of the tumor immune microenvironment [65, 66]. Collectively, the ERS-related gene signature is related to poor prognosis and a higher degree of malignancy in gliomas.

Immune ingredient quantification can be enhanced by the immune score in tumors, significantly affecting patient prognosis. In the tumor immune microenvironment, tumor purity is associated with the proportion of tumor cells [67]. Based on the risk score, glioma patients were divided into high- and low-risk subtypes. The high-risk set had a higher degree of immune infiltrate and lower tumor purity than the low-risk set. Patients with high-risk subtypes have a poor prognosis. We speculate that higher frequency mutations and higher density mutations in core pathways lead to high-risk and low-risk patient survival differences. Moreover, for immune infiltrating cells, the high-risk subtype was significantly distinct from the low-risk subtype. Performing the ssGSEA algorithm on the immune cell fraction of glioma cell subtypes, we discovered a high percentage of aDC cells, eosinophil cells, iDC macrophage cells, neutrophil cells, Th2 cells, NK cells, Th17 cells, and T cells in the high-risk subtype. NK cells had the highest proportion of infiltrating glioma lymphocytes, which is correlated with breast cancer and melanoma, indicating a prominent role for NK cells in glioma surveillance [68]. T cells are identified as a distinct functional state based on cytotoxicity or cytokine secretion responses and tissue tropisms to kill multiple brain tumor cells [53]. B cells and NK cells can be used as targets for immunotherapy [60, 69, 70]. In addition, we measured 38 immune checkpoints, and the results indicated a significant difference in most of the immune checkpoints between the high- and low-risk groups, such as PDCD1 (PD-1), CTLA4, and other checkpoints. The high-risk subtype may benefit more from immunotherapy with these checkpoints and prolong survival time. Concerning the above results, TIDE is a newly computed algorithm [71], which is believed to strongly assess the accuracy of immune checkpoint inhibitors. Our research evaluated the results from two sides of immunotherapy of high- and low-risk samples. According to calculating TIDE scores, we concluded that high-risk samples might have a better response to immune checkpoints (PD-1, CTLA4), which confirms the results above. Although immunotherapy performed some benefits on clinical glioma cancer patients, the other patients did not perform the same effects and had to adopt classical chemotherapy to cure their disease. Etoposide is a DNA damaging agent that has been used clinically both as a single agent and as a constituent of combination chemotherapy regimens for malignant brain tumor treatment [72]. We concluded that both of the groups were sensitive to five chemotherapeutic drugs (etoposide, cisplatin, erlotinib, dasatinib, lapatinib) based on the database analysis and determined that high-risk group patients had a better response to chemotherapy, which would provide a vivid view to investigators to consider searching and developing new drugs because of positive therapeutic efficacy. The above discussion implies that the heterogeneity of the tumor immune microenvironment generates different responses to immunotherapy or anticancer drugs. The ERS-related gene signature is associated with immunotherapy and chemotherapy.

In conclusion, our research identified ERS-related genes that have significant molecular and clinical characteristics of glioma. The 7-ERS-related genes can be potential prognostic and treatment biomarker sites for glioma patients. In addition, a seven-ERS-related risk model could better evaluate survival for glioma. In addition, our signature could analyze glioma patients’ immune checkpoint inhibitor response and indicate chemotherapeutic sensitivity, as well as explore new clinical therapies for glioma patients. Nonetheless, the concrete mechanism between ERS-related genes and the prognosis and immune response of glioma is unclear, and we need more experiments to explore ERS-related mechanisms in glioma.

## Supplementary Information

**Additional file 1.**

## Data Availability

The datasets generated and analyzed during the current study are available in the TCGA[https://xenabrowser.net/datapages/] and CGGA[http://www.cgga.org.cn/index.jsp] repositories.

## Abbreviations

CGGA: Chinese Glioma Genome Atlas
GTEx: Genotype-Tissue Expression
TCGA: The Cancer Genome Atlas
OS: Overall survival
UPR: Unfolded protein response
TIICs: Tumor-infiltrating immune cells
ERS: Endoplasmic reticulum stress
KM: Kaplan–Meier
ROC: Receiver operating characteristic
ICD: Immunogenic cancer cell death
TME: Tumor microenvironment
KEGG: Kyoto Encyclopedia of Genes and Genomes
GSEA: Gene set enrichment analysis
GO: Gene Ontology
ATF6: Activating transcription Factor 6
PERK1: Inositol-requiring protein-1
IDH1: Isocitrate dehydrogenase 1
CAMs: Cell adhesion molecules
HMOX1: Heme oxygenase 1
CYBB: Cytochrome b-245 beta chain
TP53: Tumor protein p53
ERN1: Endoplasmic reticulum to nucleus signaling 1
CASP8: Caspase 8
CXCL2: C-X-C motif chemokine ligand 2
PDIA5: Protein disulfide isomerase family A member 5
GDSC: Genomics of Drug Sensitivity in Cancer
TIDE: Tumor immune dysfunction and exclusion
IC50: Inhibitory concentration

## References

[1] Ferluga S, Debinski W (2014). Ephs and Ephrins in malignant gliomas. Growth Factors.

[2] Weller M, Wick W, Aldape K, Brada M, Berger M, Pfister SM, Nishikawa R, Rosenthal M, Wen PY, Stupp R (2015). Glioma. Nat Rev Dis Primers.

[3] Lapointe S. Primary brain tumours in adults. 2018;392:15. 10.1016/S0140-6736(18)30990-5. 10.1016/S0140-6736(18)30990-5 30060998

[4] Kang BR, Yang S-H, Chung B-R, Kim W, Kim Y (2016). Cell surface GRP78 as a biomarker and target for suppressing glioma cells. Sci Rep.

[5] Vom Berg J, Vrohlings M, Haller S, Haimovici A, Kulig P, Sledzinska A, Weller M, Becher B (2013). Intratumoral IL-12 combined with CTLA-4 blockade elicits T cell-mediated glioma rejection. J Exp Med.

[6] Fecci PE, Ochiai H, Mitchell DA, Grossi PM, Sweeney AE, Archer GE, Cummings T, Allison JP, Bigner DD, Sampson JH (2007). Systemic CTLA-4 blockade ameliorates glioma-induced changes to the CD4+ T cell compartment without affecting regulatory T-cell function. Clin Cancer Res.

[7] Kamran N, Kadiyala P, Saxena M, Candolfi M, Li Y, Moreno-Ayala MA, Raja N, Shah D, Lowenstein PR, Castro MG (2017). Immunosuppressive myeloid cells’ blockade in the glioma microenvironment enhances the efficacy of immune-stimulatory gene therapy. Mol Ther.

[8] Fitzmaurice C, Abate D, Abbasi N. Global Burden of Disease Cancer Collaboration. Global, regional, and national cancer incidence, mortality, years of life lost, years lived with disability, and disability-adjusted life-years for 29 cancer groups, 1990to 2017: a systemic analysis for the global burden of disease study (vol 5, pg 1749, 2019). Jama Oncology. 2020(6):444–4. 10.1001/jamaoncol.2020.0224. 10.1001/jamaoncol.2019.2996 PMC6777271 31560378

[9] He Y, Su J, Lan B, Gao Y, Zhao J (2019). Targeting off-target effects: endoplasmic reticulum stress and autophagy as effective strategies to enhance temozolomide treatment. Oncotargets and Therapy.

[10] Chen X, Cubillos-Ruiz JR (2021). Endoplasmic reticulum stress signals in the tumour and its microenvironment. Nat Rev Cancer.

[11] G. Johnson G, C. White M, Grimaldi M. (2011). Stressed to death: targeting endoplasmic reticulum stress response induced apoptosis in gliomas. Curr Pharm Des.

[12] Le Reste P-J, Avril T, Quillien V, Morandi X, Chevet E (2016). Signaling the unfolded protein response in primary brain cancers. Brain Res.

[13] Liu K, Tsung K, Attenello FJ (2020). Characterizing cell stress and GRP78 in glioma to enhance tumor treatment. Front Oncol.

[14] Markouli M, Strepkos D, Papavassiliou AG, Piperi C (2020). Targeting of endoplasmic reticulum (ER) stress in gliomas. Pharmacol Res.

[15] Wang M, Kaufman RJ (2016). Protein misfolding in the endoplasmic reticulum as a conduit to human disease. Nature.

[16] Lee HK, Xiang C, Cazacu S, Finniss S, Kazimirsky G, Lemke N, Lehman NL, Rempel SA, Mikkelsen T, Brodie C (2008). GRP78 is overexpressed in glioblastomas and regulates glioma cell growth and apoptosis. Neuro-Oncology.

[17] Ciechomska IA, Gabrusiewicz K, Szczepankiewicz AA, Kaminska B (2013). Endoplasmic reticulum stress triggers autophagy in malignant glioma cells undergoing cyclosporine A-induced cell death. Oncogene.

[18] Pyrko P, Schönthal AH, Hofman FM, Chen TC, Lee AS (2007). The unfolded protein response regulator GRP78/BiP as a novel target for increasing chemosensitivity in malignant gliomas. Cancer Res.

[19] Klawitter J, Kominsky DJ, Brown JL, Klawitter J, Christians U, Leibfritz D, Melo JV, Eckhardt SG, Serkova NJ (2009). Metabolic characteristics of imatinib resistance in chronic myeloid leukaemia cells. Br J Pharmacol.

[20] Chen D, Rauh M, Buchfelder M, Eyupoglu IY, Savaskan N (2017). The oxido-metabolic driver ATF4 enhances temozolamide chemo-resistance in human gliomas. Oncotarget.

[21] Kanzawa T, Germano IM, Komata T, Ito H, Kondo Y, Kondo S (2004). Role of autophagy in temozolomide-induced cytotoxicity for malignant glioma cells. Cell Death Differ.

[22] Nanegrungsunk D, Onchan W, Chattipakorn N, Chattipakorn SC (2015). Current evidence of temozolomide and bevacizumab in treatment of gliomas. Neurol Res.

[23] Tesniere A, Panaretakis T, Kepp O, Apetoh L, Ghiringhelli F, Zitvogel L, Kroemer G (2008). Molecular characteristics of immunogenic cancer cell death. Cell Death Differ.

[24] Krysko DV, Garg AD, Kaczmarek A, Krysko O, Agostinis P, Vandenabeele P (2012). Immunogenic cell death and DAMPs in cancer therapy. Nat Rev Cancer.

[25] Cancer Genome Atlas Research Network. Comprehensive genomic characterization defines human glioblastoma genes and core pathways. Nature. 2008;455:1061–68. 10.1038/nature07385. 10.1038/nature07385 PMC2671642 18772890

[26] Korshunov A, Ryzhova M, Hovestadt V, Bender S, Sturm D, Capper D, Meyer J, Schrimpf D, Kool M, Northcott PA (2015). Integrated analysis of pediatric glioblastoma reveals a subset of biologically favorable tumors with associated molecular prognostic markers. Acta Neuropathol.

[27] Reifenberger G, Wirsching H-G, Knobbe-Thomsen CB, Weller M (2017). Advances in the molecular genetics of gliomas - implications for classification and therapy. Nat Rev Clin Oncol.

[28] Kanehisa M, Furumichi M, Sato Y, Ishiguro-Watanabe M, Tanabe M (2021). KEGG: integrating viruses and cellular organisms. Nucleic Acids Res.

[29] Yu G, Wang L-G, Han Y, He Q-Y (2012). clusterProfiler: an R package for comparing biological themes among gene clusters. OMICS.

[30] Barbie DA, Tamayo P, Boehm JS, Kim SY, Moody SE, Dunn IF, Schinzel AC, Sandy P, Meylan E, Scholl C (2009). Systematic RNA interference reveals that oncogenic KRAS-driven cancers require TBK1. Nature.

[31] Hänzelmann S, Castelo R, Guinney J (2013). GSVA: gene set variation analysis for microarray and RNA-seq data. BMC Bioinformatics.

[32] Nagarsheth N, Wicha MS, Zou W (2017). Chemokines in the cancer microenvironment and their relevance in cancer immunotherapy. Nat Rev Immunol.

[33] Hoshida Y, Brunet J-P, Tamayo P, Golub TR, Mesirov JP (2007). Subclass mapping: identifying common subtypes in independent disease data sets. PLoS One.

[34] Geeleher P, Cox N, Huang RS (2014). pRRophetic: an R package for prediction of clinical chemotherapeutic response from tumor gene expression levels. PLoS One.

[35] Mao Y, Keller ET, Garfield DH, Shen K, Wang J (2013). Stromal cells in tumor microenvironment and breast cancer. Cancer Metastasis Rev.

[36] Tu Z, Wu L, Wang P, Hu Q, Tao C, Li K, Huang K, Zhu X (2020). N6-Methylandenosine-related lncRNAs are potential biomarkers for predicting the overall survival of lower-grade glioma patients. Front Cell Dev Biol.

[37] Xia P, Li Q, Wu G, Huang Y (2021). An immune-related lncRNA signature to predict survival in glioma patients. Cell Mol Neurobiol.

[38] Zhou Z, Huang R, Chai R, Zhou X, Hu Z, Wang W, Chen B, Deng L, Liu Y, Wu F (2018). Identification of an energy metabolism-related signature associated with clinical prognosis in diffuse glioma. Aging.

[39] Li G, Scull C, Ozcan L, Tabas I (2010). NADPH oxidase links endoplasmic reticulum stress, oxidative stress, and PKR activation to induce apoptosis. J Cell Biol.

[40] Farago N, Kocsis AK, Lovas S, Molnar G, Boldog E, Rozsa M, Szemenyei V, Vamos E, Nagy LI, Tamas G (2013). Digital PCR to determine the number of transcripts from single neurons after patch-clamp recording. Biotechniques.

[41] Jiang CC, Chen LH, Gillespie S, Wang YF, Kiejda KA, Zhang XD, Hersey P (2007). Inhibition of MEK sensitizes human melanoma cells to endoplasmic reticulum stress-induced apoptosis. Cancer Res.

[42] Maines MD (1988). Heme oxygenase: function, multiplicity, regulatory mechanisms, and clinical applications. FASEB J.

[43] Berberat PO, Dambrauskas Z, Gulbinas A, Giese T, Giese N, Kunzli B, Autschbach F, Meuer S, Buchler MW, Friess H (2005). Inhibition of heme oxygenase-1 increases responsiveness of pancreatic cancer cells to anticancer treatment. Clin Cancer Res.

[44] Gandini NA, Fermento ME, Salomon DG, Obiol DJ, Andres NC, Zenklusen JC, Arevalo J, Blasco J, Lopez Romero A, Facchinetti MM (2014). Heme oxygenase-1 expression in human gliomas and its correlation with poor prognosis in patients with astrocytoma. Tumor Biol.

[45] Ogata M, Hino S, Saito A, Morikawa K, Kondo S, Kanemoto S, Murakami T, Taniguchi M, Tanii I, Yoshinaga K (2006). Autophagy is activated for cell survival after endoplasmic reticulum stress. Mol Cell Biol.

[46] Tay KH, Luan Q, Croft A, Jiang CC, Jin L, Zhang XD, Tseng H-Y (2014). Sustained IRE1 and ATF6 signaling is important for survival of melanoma cells undergoing ER stress. Cell Signal.

[47] Zeng L, Lu M, Mori K, Luo S, Lee AS, Zhu Y, Shyy JY-J (2008). ATF6 modulates SREBP2-mediated lipogenesis (vol 23, pg 950, 2004). EMBO J.

[48] Chibon F (2013). Cancer gene expression signatures - the rise and fall?. Eur J Cancer.

[49] Xu Y, Li R, Li X, Dong N, Wu D, Hou L, Yin K, Zhao C (2020). An autophagy-related gene signature associated with clinical prognosis and immune microenvironment in gliomas. Front Oncol.

[50] Xu S, Tang L, Liu Z, Yang K, Cheng Q (2021). Bioinformatic analyses identify a prognostic autophagy-related Long non-coding RNA signature associated with immune microenvironment in diffuse gliomas. Front Cell Dev Biol.

[51] Zhuo S, Chen Z, Yang Y, Zhang J, Tang J, Yang K (2020). Clinical and biological significances of a Ferroptosis-related gene signature in glioma. Front Oncol.

[52] Zhang Z, Chen J, Huo X, Zong G, Huang K, Cheng M, Sun L, Yue X, Bian E, Zhao B (2021). Identification of a mesenchymal-related signature associated with clinical prognosis in glioma. Aging.

[53] Zhu Y, Qiu P, Ji Y (2014). TCGA-assembler: open-source software for retrieving and processing TCGA data. Nat Methods.

[54] Yang D, Li R, Wang H, Wang J, Li Y, Wang H, Wang W, Liu Z (2018). Clinical significance of tumor necrosis factor receptor 2 in middle and lower thoracic esophageal squamous cell carcinoma. Oncol Lett.

[55] Yoon J, Chung YE, Lim JS, Kim M-J (2019). Quantitative assessment of mesorectal fat: new prognostic biomarker in patients with mid-to-lower rectal cancer. Eur Radiol.

[56] Sicari D, Fantuz M, Bellazzo A, Valentino E, Apollonio M, Pontisso I, Di Cristino F, Dal Ferro M, Bicciato S, Del Salf G (2019). Mutant p53 improves cancer cells’ resistance to endoplasmic reticulum stress by sustaining activation of the UPR regulator ATF6. Oncogene.

[57] Harris SL, Levine AJ (2005). The p53 pathway: positive and negative feedback loops. Oncogene.

[58] Amberger-Murphy V (2009). Hypoxia helps glioma to fight therapy. Curr Cancer Drug Targets.

[59] Song Y, Zheng S, Wang J, Long H, Fang L, Wang G, Li Z, Que T, Liu Y, Li Y (2017). Hypoxia-induced PLOD2 promotes proliferation, migration and invasion via PI3K/Akt signaling in glioma. Oncotarget.

[60] Dietrich P-Y, Dutoit V, Thang NNT, Walker PR (2010). T-cell immunotherapy for malignant glioma: toward a combined approach. Curr Opin Oncol.

[61] Ahmed N, Salsman VS, Kew Y, Shaffer D, Powell S, Zhang YJ, Grossman RG, Heslop HE, Gottschalk S (2010). HER2-specific T cells target primary glioblastoma stem cells and induce regression of autologous experimental tumors. Clin Cancer Res.

[62] Healy SJM, Gorman AM, Mousavi-Shafaei P, Gupta S, Samali A (2009). Targeting the endoplasmic reticulum-stress response as an anticancer strategy. Eur J Pharmacol.

[63] Kim I, Xu W, Reed JC (2008). Cell death and endoplasmic reticulum stress: disease relevance and therapeutic opportunities. Nat Rev Drug Discov.

[64] Schröder M, Kaufman RJ (2005). The mammalian unfolded protein response. Annu Rev Biochem.

[65] Jhaveri N, Chen TC, Hofman FM (2016). Tumor vasculature and glioma stem cells: contributions to glioma progression. Cancer Lett.

[66] Quezada C, Torres A, Niechi I, Uribe D, Contreras-Duarte S, Toledo F, San Martin R, Gutierrez J, Sobrevia L (2018). Role of extracellular vesicles in glioma progression. Mol Asp Med.

[67] Zhang C, Cheng W, Ren X, Wang Z, Liu X, Li G, Han S, Jiang T, Wu A (2017). Tumor purity as an underlying key factor in glioma. Clin Cancer Res.

[68] Holl EK, Frazier VN, Landa K, Beasley GM, Hwang ES, Nair SK (2019). Examining peripheral and tumor cellular Immunome in patients with Cancer. Front Immunol.

[69] Golan I, Rodriguez de la Fuente L, Costoya JA. NK Cell-Based Glioblastoma Immunotherapy Cancers (2018) 10:522. doi:10.3390/cancers10120522. 10.3390/cancers10120522 PMC6315402 30567306

[70] Sedgwick AJ, Ghazanfari N, Constantinescu P, Mantamadiotis T, Barrow AD (2020). The role of NK cells and innate lymphoid cells in brain Cancer. Front Immunol.

[71] Jiang P, Gu S, Pan D, Fu J, Sahu A, Hu X, Li Z, Traugh N, Bu X, Li B (2018). Signatures of T cell dysfunction and exclusion predict cancer immunotherapy response. Nat Med.

[72] Sawada M, Nakashima S, Banno Y, Yamakawa H, Hayashi K, Takenaka K, Nishimura Y, Sakai N, Nozawa Y (2000). Ordering of ceramide formation, caspase activation, and Bax/Bcl-2 expression during etoposide-induced apoptosis in C6 glioma cells. Cell Death Differ.

