# Supplementary material for: Identification of an endoplasmic reticulum stress-related signature associated with clinical prognosis and immune therapy in glioma
Source: BMC Neurol. 2022 May 25;22:192. doi: 10.1186/s12883-022-02709-y (PMC9131635; doi:10.1186/s12883-022-02709-y)
Supplement: Supplementary file 1 — Additional file 1. [file 12883_2022_2709_MOESM1_ESM.docx]

**Supplementary Material**


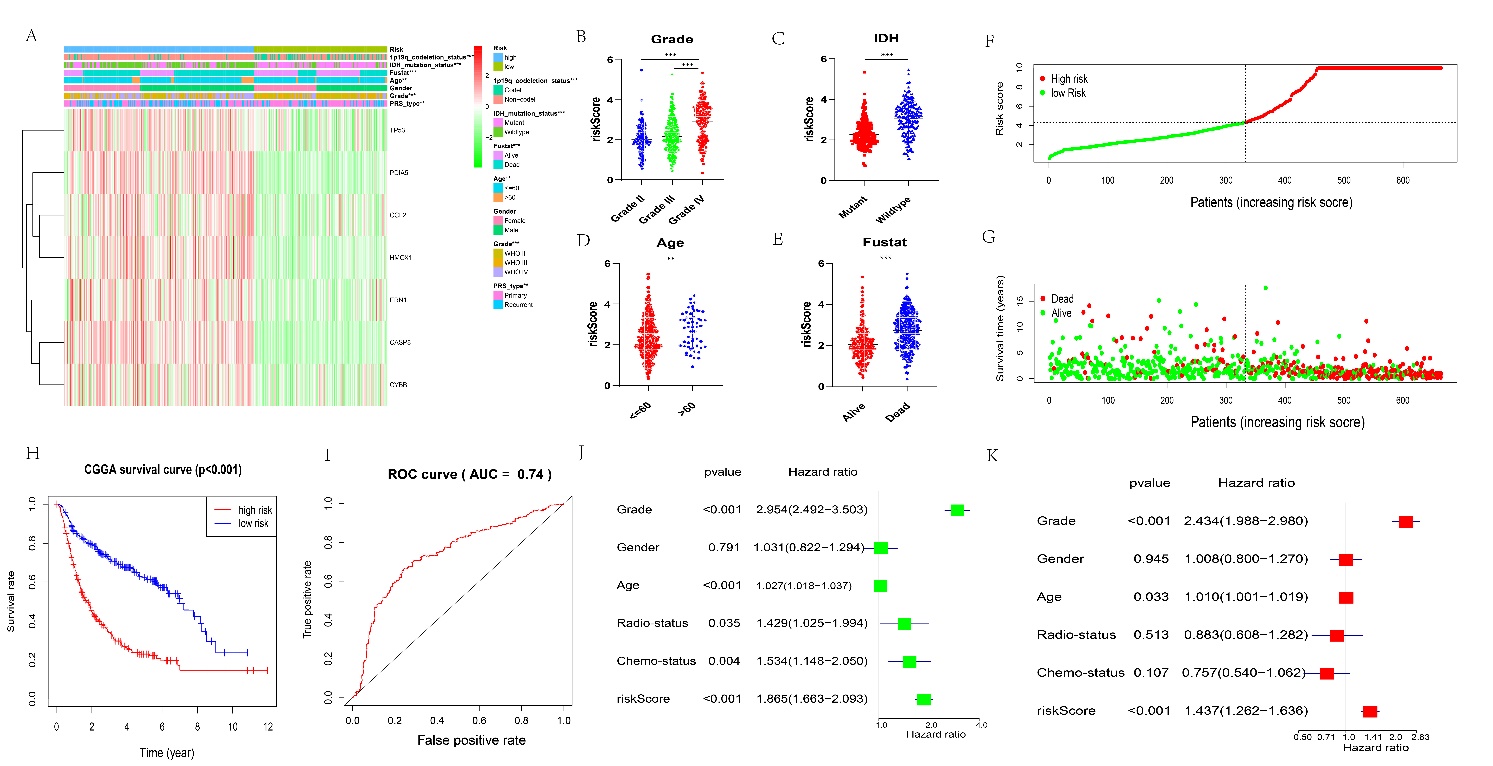


**Supplementary Figure 1 |** Verification of the 7-gene risk model in CGGA datasets. (**A**) The heatmap showed the expression difference of seven ERS-related genes between the high risk subtype and low risk subtype. **(B**-**E)** The WHO grade **(B)**, IDH status **(C)**, age **(D)**, fustat **(E)** stratify the CGGA dataset, and the distribution of risk scores is shown. (**F**) The risk curve represents the risk score and distribution of 619 cases from the CGGA database. (**G**) The survival status graph shows the difference in survival time of 619 cases from the CGGA database (each point represents a sample, B-C). (**H**) Kaplan-Meier algorithm among the high-risk set and low-risk set from the CGGA database. (**I**) ROC algorithm indicates the specificity and sensitivity to predict survival time with the ERS-related signature from the CGGA database. (**J**) Univariate Cox regression algorithm of clinical and pathological features for survival rate from the CGGA dataset. (**K**) Multivariate Cox regression algorithm of clinical and pathological features for survival rate from the CGGA dataset. **P <0.01; ***P <0.001.


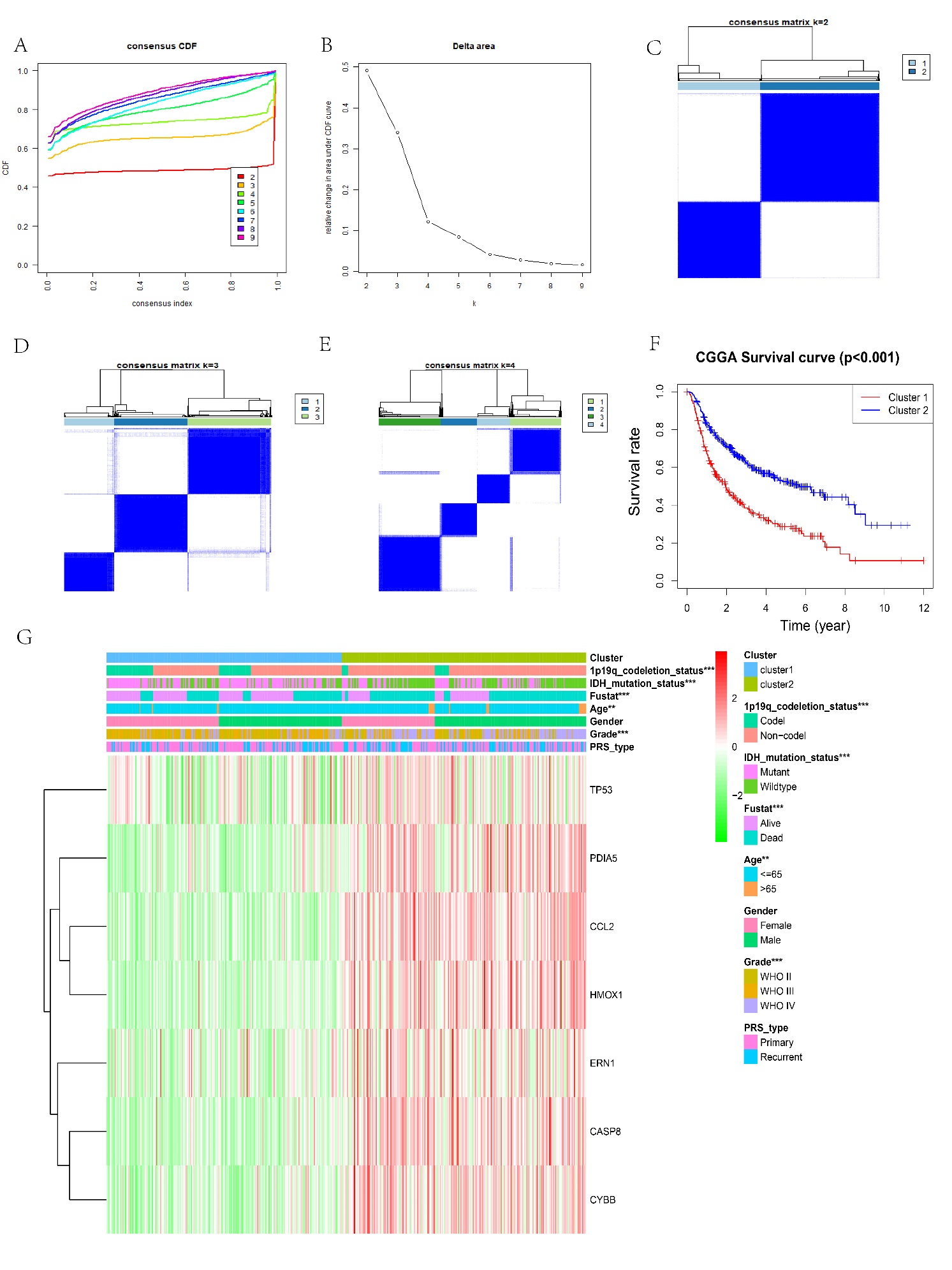


**Supplementary Figure 2 |** Stratification and verification of glioma based on 7-ERS-related gene signature in the CGGA cohort. (**A**) The Relative changes in the area under the CDF curve (k = 2 to 9) in the testing set. (**B**) Consensus cluster CDF from k=2 to 9 in the testing set. (**C-E**) Consensus cluster algorithm of 1018 CGGA data for k = 2 to k = 4. (**F**) The cluster 1 and cluster 2 patients for survival curve based on CGGA clinical data. (**G**) Heatmap of ERS-related genes among two clusters based on the CGGA dataset to verify consensus clustering based on the TCGA. CDF, cumulative distribution function; **P <0.01; ***P <0.001.


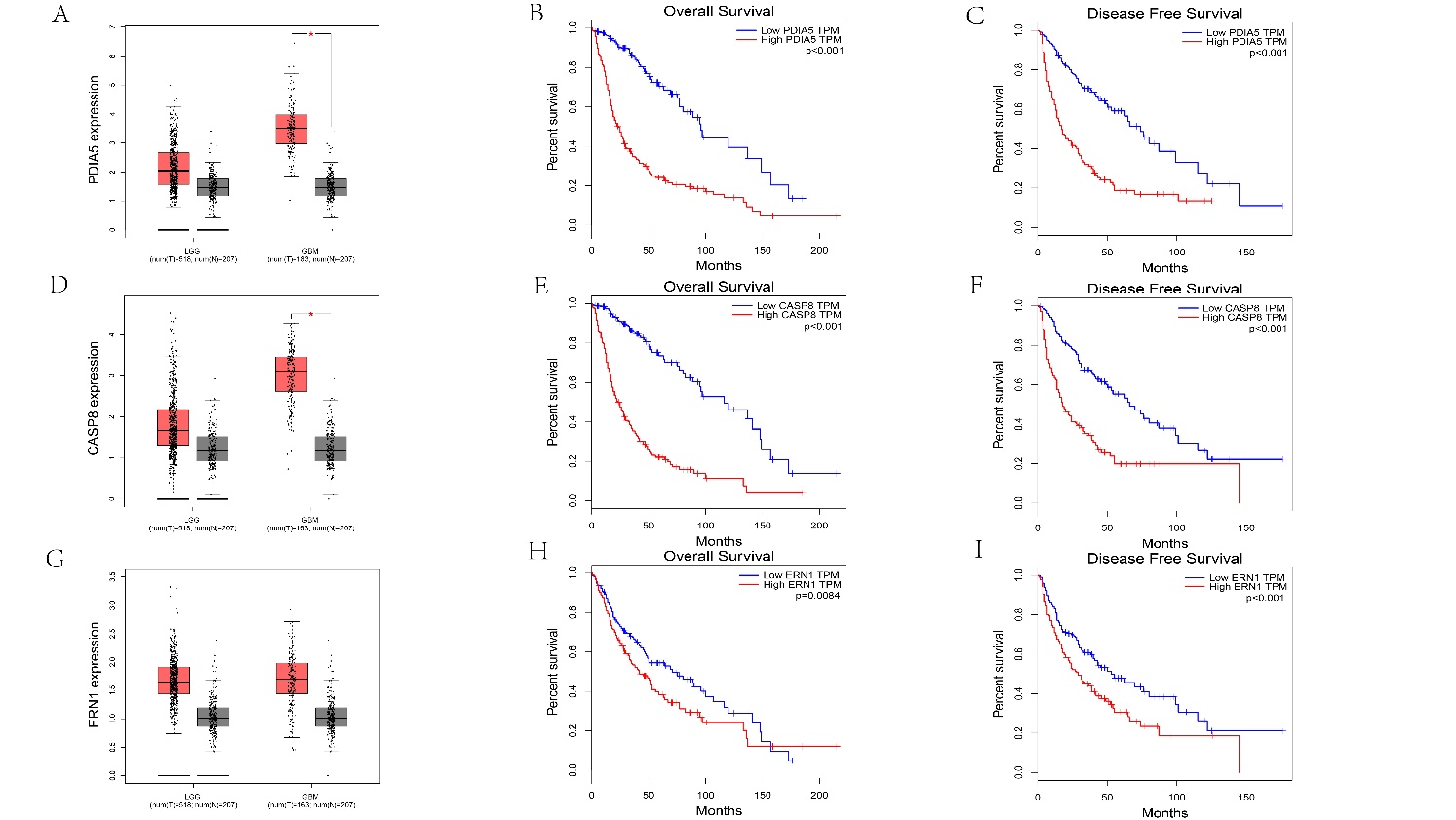


**Supplementary Figure 3 |** PDIA5, CASP8, and ERN1 gene expression differences and survival rate differences. (**A**) Differences of PDIA5 expression among the tumor and the normal group from the GTEX and TCGA database. (**B-C**) Kaplan Meier overall survival and disease-free survival curves showed the correlation among PDIA5 expression and survival rate based on the TCGA dataset. (**D**) Differences of CASP8 expression among the tumor and the normal group from the GTEX and TCGA database. (**E-F**) Kaplan Meier overall survival and disease-free survival curves showed the correlation among CASP8 expression and survival rate based on the TCGA dataset. (**G**) Differences of ERN1 expression among the tumor and the normal group from the GTEX and TCGA database. (**H-I**) Kaplan Meier overall survival and disease-free survival curves showed the correlation among ERN1 expression and survival rate based on the TCGA dataset. Ns: no significance; ***P<0.001.
